# Why under five children are stunted in Pakistan? A multilevel analysis of Punjab Multiple indicator Cluster Survey (MICS-2014)

**DOI:** 10.1186/s12889-020-09110-9

**Published:** 2020-06-17

**Authors:** Tahir Mahmood, Faisal Abbas, Ramesh Kumar, Ratana Somrongthong

**Affiliations:** 1 Department of Economics, University of Chitral, Chitral, Pakistan; 2 American University of Beirut, Beirut, Lebanon; 3 Department of Economics, School of Social Sciences and Humanities (S3H), National University of Sciences and Technology (NUST), Islamabad, Pakistan; 4 Health Systems & Policy Department, Health Services Academy, Islamabad, Pakistan; 5 College of Public Health Sciences, Chulalongkorn University, Bangkok, Thailand

**Keywords:** Undernutrition, Stunting, Child health, Pakistan, Punjab, Multilevel models, Multiple indicators cluster survey

## Abstract

**Background:**

Pakistan is facing a serious problem of child under-nutrition as about 38% of children in Pakistan are stunted. Punjab, the largest province by population and contributes high gross domestic product (GDP) share in economy has reported 27% moderately and 10% severely stunted children of less than 5 years. Thus, this study aims at examining the determinants of stunting (moderate and severe) at different level of hierarchy empirically in Punjab province of Pakistan.

**Methodology:**

Data for this study is coming from Punjab Multiple Indicators Cluster Survey (MICS-2014), used two-stage, stratified cluster sampling approach. Sub-national level data covering urban and rural areas were used for this study consists of 25,067 children less than 5 year’s ages, from nine administrative divisions and 36 districts of Punjab province of Pakistan. Descriptive statistics and multilevel hierarchical models were estimated. Multilevel data analyses have an advantage because it provides robust standard error estimates and helps in finding variation in the data at various levels.

**Results:**

Punjab has a stunting prevalence of about 27% moderately and 10% severely stunted children of less than 5 years. The results depict that increasing the age of the child, increasing birth order, illiterate mothers and fathers, lack of sanitation facilities and being poor are associated significantly with the likelihood of moderate and severe stunting. Surprisingly, there is a gender bias in stunting in Punjab, Pakistan and being a girl child is more likely associated with moderate and severe stunting, which shows the patriarchal nature of the society and a substantial prevalence of gender bias in household resource allocations.

**Conclusion:**

This outcome of our analysis points towards targeting not only households (focus on girls) but also their families and communities.

## Background

One out of five children under 5 year’s age is reported stunted and contributes half of the child mortality in the world [1].. Globally, more than half (56%), 87 million of the total stunted children are living in South Asia alone. A stunted child is physically short of their age and thus fails to grow cognitively. This condition of undernourishment of a child undermines their potential of life from its very manifestation and affects over the life cycle [2]. Under-nutrition leads to child morbidity and mortality, poor physical and cognitive development, poor school performance, reduced capacity to work even later in life, thus loss of productivity and wages [3–5]. Therefore, under-nutrition is one of the most pressing challenges of the contemporary era due to its long-lasting and detrimental consequences [6].

Moreover, inadequate nutrition of the mother during pregnancy and in the first 2 years of a child’s life is an important window of opportunity to realize a child’s full potential of life. If this window of opportunity is missed could cause irreversible and retarded growth, impaired learning and reduced work performance [2]. Stunting refers to poor child health status due to multiple factors like; physical development, cognitive abilities and the environment [7–11]. Stunting is the form of undernutrition (reduced height for age) evaluated by using anthropometric indices as a proxy measure according to WHO child growth standards [12, 13]. After India, Pakistan has the highest number of stunted children in South Asia [14–16]. Moreover, malnutrition is widespread in Pakistan among all age groups and the progress is not promising in the last decades [17, 18].

In Punjab, stunting prevalence in the year 2011 was 36% and in 2014, it is reduced to 33.5%, this prevalence rate of stunting is alarming for the largest and most developed province of the country. Punjab contributes more than 52% to the national income and about 56% of the population of the country resides in this province. Given the severe prevalence of stunting in one of the largest province of Pakistan, this study investigates in detail the prevalence and determinants of stunting in Punjab as an important research question. Moreover, it is imperative to analyze the factors, i.e., immediate (Child level characteristics), underlying (mother, father, household and community level characteristics) and basic (i.e., areas of residence, the region of residence and regional food security status) associated with stunting in Punjab, Pakistan [13].

The main factors associated with stunting according to literature are; multiple births, low birth-weight, maternal education, household wealth, maternal body mass index and the short birth interval [19]. Similarly, previous studies shows that an individual and community-level factors are important determinants of childhood stunting and confirm that 37% of stunting is due to community-level factors in their study [20, 21]. Apart from child specific characteristics and parental characteristics, the importance of water, sanitation and hygiene (WASH) have been recognized regarding health of infants and young children, particularly when the process of stunting is concentrated during the first 2 years of life [10].

Recent studies show that the poor diet of children in the first year of life, poor nutritional status of a mother during and after pregnancy, poor sanitation practices, food insecurity and poverty, and social inequalities are significant drivers of childhood stunting [22–24]. In addition, child nutritional status is commonly influenced by factors such as adequate health services and/or lack thereof, nutritional and social services facilities at community/village level and access to these facilities [25–28]. Another study found that safe water access, education of women, gender equality, and food availability are the main drivers in reducing stunting [1].

Although, literature on Pakistan is rather scant [18]. There are attempts to investigate the determinants of under-nutrition not only at the national level but also at the micro-geographic level [29–34]. A study from Sindh province also pinpoints mother illiteracy, low income, and overcrowding as significant risk factors of stunting [30]. Additionally, education among rural mothers could reduce stunting, as being female are more likely to be stunted and underweight compared to male in these areas [31]. Contrary to this assertion, for Punjab, a study using MICS 2011 data explored that female headed-household (a measure of female empowerment) had 26% lower odds of childhood stunting [34].

All these research studies have focused on employing either ordinary least square (OLS) or binary logistic regression analyses. As standard OLS may over or under-estimate the coefficient of correlates at different points across the distribution of stunting prevalence, thus, bias the estimates. These hierarchies have vital information to explore for better and targeted policy formulation. Therefore, this study uses a multilevel hierarchical modeling approach to unravel the variation at household, community and the district level that is not explored in the case of Pakistan at the micro-geographic level. This analysis will set up the stage for policy level debate and provide the data-driven information for public policy design. This study is first to analyze the determinants of stunting in Punjab Pakistan using MICS-2014, which includes more than 31,000 children of less than 5 years [13].

## Methods

The Multiple Indicator Cluster Survey (MICS) is an international household survey program developed by United Nations Children’s Fund (UNICEF) to collect comparable estimates of more than 125 key indicators that are used to assess the situation of children and women, in Punjab Pakistan. The MICS consists of all the households and their members in all urban and rural areas of Punjab, which is divided into 9 administrative divisions and 36 districts. A two-stage, stratified cluster sampling approach was used for the selection of the survey sample. The first-stage selection unit is an enumeration and the village in urban and rural areas. From each of the first-stage sample units, a sample of 20 households selected both in rural and urban areas. Households were considered as secondary sampling units (SSUs) for urban and rural domains. The entire sample of households (SSUs) was drawn from 2050 Primary Sampling Units (PSUs), out of which 774 were urban and 1276 were rural, adopting a systematic sampling technique with a random start. The final recommended allocation is 2050 clusters with 20 households in each, giving a total sample of 41,000 households and the response rate is almost 98% across Punjab province. The detail on MICS Punjab 2014 is provided elsewhere [13].

The current study investigates the determinants of stunting of under five years of children. For this purpose, after cleaning the data, we end up with 25,066 children of under 5 year of age.

### Dependent variables

Stunting (i.e., moderate and severe) is used as a binary variable with not stunted being coded as zero while being stunted coded as one, similar is the case with severe stunted. Stunted is measured as moderate if the height for age z score of a child less than 5 years is less than minus 2 standard deviation from WHO reference growth standards and it is severe if z score is less than minus 3 standard deviation [35].

### Independent variables

Individual and household level characteristics include; the age of a child in months and is categorized into (0–5 months, 6–11 months, 12–23 months, 24–35 months, 36–47 months and 48–59 months). We divided the first year of the child into two categories because for the first 6 months, a child is exclusively breast-fed or having milk only as his (her) diet. Moreover, between 6 and 12 months, he/she is starting solid food as well. Birth order of the child, this variable is a rank number of children born to women age 15–49 years and categorized into; first born, second born and third or after born having age less than 5 years. The gender of a child is either boy or girl. The size of the child at birth is divided into three categories, i.e. large (combining very large and larger than average birth size category), average and small (combining smaller than average and very small category).^1^ Diarrhea, whether a child had diarrhea in the last 2 weeks (15 days), is a binary variable (Yes = 1; zero otherwise). Breastfeeding is a binary variable if a child is ever breastfed *(Yes = 1)* and *(No = 0)*.

Parental education is categorized as; no education (less than 1 year), primary (1–5 years completed), middle (6–8 years of a complete education), secondary (9–10 years of a complete education) and higher (11+ years of complete education including professional or university degrees). Mother’s age is a categorical variable; less than 18 years, 18–24 years, 25–35 years and 35+ years. Postnatal care is proxies by whether a Lady Health Worker (LHW) visited the household in the past 3 months and is a binary variable. This variable captures the knowledge and information exchange that is positively associated with child health outcomes. Antenatal care visits (ANC visits) are the number of times a woman received antenatal care during pregnancy and is coded as; less than 4 times as recommended by WHO and 4 times or higher times. Place of delivery where a woman gives birth to her child is coded as home delivery if the birth occurred at her own home or another home and institutional delivery if the delivery occurred at either public or private hospital or maternity care or basic/primary health care center. Household size includes the number of household members in a household and is categorized into; 3–4 household members, 5–6 household members which is an average size of a family in Punjab, 7–8 household members and more than 8 household members to capture the effect of a very large family on child health outcomes.

Ethnicity is asking about the mother tongue of the head of the household and is categorized into; Urdu, Punjabi, Saraiki and Others. Place of residence is a binary variable and coded as *urban = 1* and *rural = 0*. Similarly, the sanitation facility is about the toilet facility a household possesses. It has been divided into two categories, according to the WHO definition. The first category is *improved sanitation facility*, which includes sewer connections, septic system connection, pour-flush latrines, ventilated improved pit latrines and pit latrines with a slab or covered pit. The second category is *unimproved sanitation facility,* which includes pit latrines without slabs or platforms or open-pit hanging latrines bucket latrines, open defecation in fields, forests and/or bushes. Water treatment (boiling water before drinking) is asking a household what household do to make water safer for drinking and is a binary variable coded as *treated = 1* and *untreated = 0*.

Regional characteristics include districts wise food insecurity index and administrative divisions of the province of Punjab. The food insecurity index is taken from the SDPI report and this index is at the district level. All three composite indicators (availability, access and food absorption) were aggregated to determine the food insecurity level of the districts in Pakistan. The food insecurity indicator was not the simple sum of all composite indicators, but rather a principal component analysis (PCA) was used [22, 24, 36]. The food insecurity index is divided into three categories. (1) Food Insecure (0.53–0.71), (2) Vulnerable (0.72–0.80, 3) Food Secure (0.81–0.93). The measure of food insecurity helps in better understanding the variation at sub national level (district); hence this variable is included at the district level. Furthermore, the Punjab province is divided into nine administrative divisions; Rawalpindi, Bahawalpur, D. G. Khan, Faisalabad, Gujranwala, Lahore, Multan, Sahiwal and Sargodha.

### Data analysis

The simultaneous analysis of two variables is captured through bivariate analysis. It explores whether there exists a significant difference between the variables or not. This study presents the prevalence of moderate and severe childhood stunting in percentages at various levels of covariates, the level of significance of each variable with that of the response variable set at *P-value < 0.05*. In addition, the current study employs three-level random intercept multilevel hierarchical models. Many kinds of data, including observational data, have a hierarchical or clustered structure. For example, children with the same parents tend to be more alike in their physical attributes, social attitudes, and mental characteristics than children are chosen randomly. Multilevel models recognize the existence of data hierarchies by varying the residual components at each level in the hierarchy [37]. In a multilevel model, the groups in the sample are treated as a random sample, where it is not possible in the fixed-effect model. For instance, if we use ordinary regression analysis in finding factors associated with stunting, we assume that for all children, the outcomes (in this case, moderate and severe stunting) are independent. Nevertheless, in our analysis, more than one child per family is included so that outcomes for siblings may be correlated, especially if the nutritional status is dependent on characteristics common to a family such as the quality of parental care, the sanitary conditions within the household etc. Because of these household effects, we would expect stunting for children in the same household or community to be more alike than stunting for children from different households or communities [36]. A simple three-level model without the explanatory variables, which is called unconditional model is written as: *Stunting*_*ijk*_ = *b*_0_ + *v*_*k*_ + *u*_*jk*_ + *e*_*ijk*_ Where *Stunting*_*ijk*_ is the observed value of being *(Stunting*_*ijk*_*= 1)* both moderately and severely stunted and being non-stunted *(Stunting*_*ijk*_*= 0)* child under-five in household *i*, in community *j* and district *k*.

Several approaches are available to interpret variance components in multilevel models; we are considering here the; variance partition coefficients *(VPCs)* and intraclass correlation coefficients *(ICCs).* The *VPC* statistics report the proportion of the response variance that lies at each level of the model hierarchy. Thus, the total variance is simply the sum of the three separate variance components. The *ICC* measures the expected degree of similarity between responses within a specific level.

As the OR sometimes does not give a good approximation and does not provide information about the strength of the association (effect size), therefore, this study also calculates an important measure of effect size known as Cohen’s *d.* Cohen suggested a threshold that *d* = 0.2 be considered a small effect size, d = 0.5 represents a ‘medium’ effect size and d = 0.8 a ‘large’ effect size [38]. This indicates if the mean difference between the two groups is less than 0.2, the effect size is trivial even though the OR is significant. The OR of 1.68, 3.47, and 6.71, are equivalent to *Cohen’s d* = 0.2 (small), 0.5 (medium) and 0.8 (large) respectively at 1% level of significance. At 5% level, the corresponding reference points are 1.52, 2.74, and 4.72. At 10% level, corresponding reference points are 1.46, 2.49, and 4.14, respectively. The transformation of OR is done, according to Cohen [39].

## Results

### Bivariate analysis results

Table 1 presents the prevalence of moderate and severe childhood stunting in percentages at various levels of covariates, the level of significance of each variable with that of the response variable set at *P-value < 0.05*. A little more than a quarter (27.4%) of children are moderately stunted and one-tenth (10.4%) is severely stunted in Punjab, Pakistan. Furthermore, 31.5% of children are moderately stunted between the age group of 36 months to 59 months, whereas about 11.5% are severely stunted in the same age group. The figure for 0–11 months is 15%t moderately stunted, while 5.5% severely stunted children. Thus, child age is an important and significant predictor of health outcomes. Childbirth order is a significant risk factor for child stunting outcomes. For example, if a child birth order is 3 or above, the child has more likelihood of being stunted (30%) and severely stunted (11%). Whereas a little more than one-fourth (27%) of first-born are likely to be stunted and one-tenth (10%) likely to be severely stunted. Of note, 27% of boys and 28% of girls are moderately stunted, whereas 10% of boys and 11% of girls are severely stunted. Girls are facing significantly higher burden of moderate and severe stunting. Child size at birth is also an important predictor; for example, children born larger than average size are (21%) moderately stunted and (8%) severely stunted, but those born with smaller than average birth size are 36% moderately and 15% severely stunted. Almost 24% of children who breastfeed are moderately and about 9% severely stunted compared to 25%; those who are not breast-feed are moderately stunted and 12% severely stunted. Children having diarrhea are more likely to be stunted (31%) compared to those who are not having diarrhea (27%).

**Table 1:** Prevalence of moderate and severe childhood stunting at various levels of independent variables in Punjab, Pakistan (Punjab MICS-2014) (*n* = 25,066)

Variables	Stunting (Moderate SD < − 2)			Stunting (Severe SD < − 3)		
	Total	Yes N (%)	No N (%)	Total	Yes N (%)	No N (%)
Total (%)	25,066	6865 (27.4)	18,201 (72.6)	25,066	2602 (10.4)	22,464 (89.6)
***Individual and Household Level characteristics***						
**Age Group**	**< 0.001**			**< 0.001**		
0–5 months	2177	247 (11)	1930 (89)	2177	99 (5)	2078 (95)
6–11	2767	531 (19)	2236 (81)	2767	157 (6)	2609 (94)
12–23	4827	1484 (31)	3343 (69)	4827	569 (12)	4258 (88)
24–35	4838	1266 (26)	3572 (74)	4838	478 (10)	4360 (90)
36–47	5373	1785 (33)	3588 (76)	5373	657 (12)	4716 (88)
48–59	5082	1549 (30)	3533 (70)	5082	641 (13)	4441 (87)
**Birth Order**	**0.04**			**0.023**		
1st	16,391	4415 (27)	11,976 (73)	16,435	1641 (10)	14,794 (90)
2nd	7323	2046 (28)	5277 (68)	7323	816 (11)	6507 (89)
3+	1351	403 (30)	948 (70)	1351	144 (11)	1207 (89)
**Gender of Child**	**0.024**			**< 0.001**		
Boy	12,712	3384 (27)	9328 (73)	12,712	1231 (10)	11,481 (90)
Girl	12,354	3481 (28)	8873 (68)	12,354	1371 (11)	10,983 (89)
**Size at Birth**	**0.001**			**0.001**		
Large	1052	218 (21)	834 (79)	1052	80 (8)	972 (92)
Average	8862	2281 (26)	6581 (74)	8862	846 (10)	8016 (90)
Small	2633	938 (36)	1695 (64)	2633	406 (15)	2227 (85)
**Diarrhea**	**< 0.001**			**< 0.001**		
Yes	4391	1344 (31)	3047 (69)	4391	556 (13)	3835 (87)
No	20,676	5522 (27)	15,154 (73)	20,675	2046 (10)	18,629 (90)
**Breast Feeding**	**< 0.001**			**< 0.001**		
Yes	13,950	3365 (24)	10,585 (76)	13,949	1226 (9)	12,723 (91)
No	703	179 (25)	524 (75)	703	81 (12)	622 (88)
**Mother Education**	**< 0.001**			**< 0.001**		
Noeducation	12,270	4428 (36)	7842 (64)	12,270	1861 (15)	10,409 (85)
Primary	4517	1149 (25)	3368 (75)	4517	367 (8)	4150 (92)
Middle	2481	508 (20)	1973 (80)	2481	156 (6)	2325 (94)
Secondary	3101	529 (17)	2572 (83)	3101	156 (5)	2945 (95)
Higher	2696	249 (9)	2447 (91)	2696	63 (2)	2633 (98)
**Mother Age**	**0.042**			**0.305**		
< 18	1696	499 (29)	1197 (71)	1696	185 (11)	1511 (89)
18–24	5148	1323 (26)	3825 (74)	5148	495 (10)	4653 (90)
25–35	13,893	3810 (27)	10,083 (73)	13,893	1428 (10)	12,465 (90)
36+	4327	1232 (28)	3095 (72)	4327	492 (11)	3835 (89)
**Antenatal Care**	**< 0.001**			**< 0.001**		
1–4 visits	6169	1725 (28)	4444 (72)	6169	625 (10)	5544 (90)
5+ visits	3914	718 (18)	3196 (82)	3914	253 (6)	3661 (94)
**Postnatal Care**	**0.723**			**0.431**		
Yes	4764	1315 (28)	3449 (72)	4764	517 (11)	4247 (89)
No	7703	2100 (27)	5603 (73)	7703	799 (10)	904 (90)
**Child Delivery**	**< 0.001**			**< 0.001**		
Home	5452	1884 (35)	3568 (65)	5452	773 (14)	4679 (86)
Hospitals	7150	1567 (22)	5583 (78)	7150	562 (8)	6588 (92)
**Father Education**	**< 0.001**			**< 0.001**		
Noeducation	7335	2814 (38)	4521 (62)	7335	1217 (17)	6118 (83)
Primary	4536	1419 (31)	3117 (69)	4536	524 (12)	4012 (88)
Middle	4138	1024 (25)	3114 (75)	4138	326 (8)	3812 (92)
Secondary	5507	1144 (21)	4363 (79)	5507	391 (7)	5116 (93)
Higher	3548	463 (13)	3085 (87)	3548	144 (4)	3405 (96)
**WealthIndex**	**< 0.001**			**< 0.001**		
Poor	11,167	4155 (37)	7012 (63)	11,167	1770 (16)	9397 (84)
Middle	4915	1218 (25)	3697 (75)	4915	395 (8)	4520 (92)
Rich	8983	1491 (17)	7492 (83)	8983	436 (5)	8547 (95)
**Household Size**	**< 0.001**			**< 0.002**		
3–4 members	2873	705 (25)	2168 (75)	2873	250 (9)	2623 (91)
5–6 members	7179	1983 (28)	5196 (68)	7179	774 (11)	6405 (89)
7–8 members	6177	1838 (30)	4339 (70)	6177	706 (11)	5471 (89)
8+ members	8836	2339 (26)	6497 (74)	8836	871 (10)	7965 (90)
***Community Level Characteristics***						
**Ethnicity**	**< 0.001**			**< 0.001**		
Urdu	1267	243 (19)	1024 (81)	1267	76 (6)	1191 (94)
Punjabi	16,141	3967 (25)	12,174 (75)	16,141	1332 (8)	14,808 (92)
Saraikai	6040	2139 (35)	3901 (65)	6040	973 (16)	5067 (84)
Others	1617	515 (32)	1102 (68)	1617	221 (13)	1396 (87)
**Region**	**< 0.001**			**< 0.001**		
Urban	7842	1620 (21)	6222 (79)	7842	538 (7)	7304 (93)
Rural	17,224	5245 (30)	11,979 (70)	17,224	2063 (12)	15,161 (88)
**Sanitation**	**< 0.001**			**< 0.001**		
Improved	17,903	4185 (23)	13,718 (77)	17,903	1429 (8)	16,474 (92)
Unimproved	7163	2679 (37)	4484 (63)	7163	1173 (16)	5990 (84)
**WaterFacilities**	**< 0.001**			**< 0.001**		
Improved	23,613	6584 (28)	17,029 (72)	23,613	2507 (11)	21,106 (89)
Unimproved	1453	281 (19)	1172 (81)	1453	94 (7)	1359 (93)
**Water Treatment**	**< 0.001**			**< 0.001**		
Treated	1524	228 (15)	1296 (85)	1425	82 (5)	1443 (95)
Untreated	23,542	6638 (28)	16,904 (72)	23,524	2521 (11)	21,023 (89)
***Regional LevelCharacteristics***						
**Food InsecurityIndex (FII)**	**< 0.001**			**< 0.001**		
Insecure	2662	1035 (39)	1626 (61)	2662	482 (18)	2179 (82)
Vulnerable	8875	2598 (29)	6277 (71)	8875	1016 (11)	7859 (89)
Secure	13,529	3232 (24)	10,297 (76)	13,529	1104 (8)	12,425 (92)
**Division**	**< 0.001**			**< 0.001**		
Rawalpindi	1909	312 (16)	1597 (84)	1909	86 (5)	1823 (95)
Bahawalpur	2930	1016 (35)	1914 (65)	2930	432 (15)	2498 (85)
D.G Khan	2822	1112 (39)	1710 (61)	2822	528 (19)	2294 (81)
Faisalabad	3115	758 (24)	2357 (76)	3115	272 (9)	2843 (91)
Gujranwala	3234	727 (22)	2507 (78)	3234	235 (7)	2999 (93)
Lahore	4416	1093 (25)	3323 (75)	4416	378 (9)	4038 (91)
Multan	2853	823 (29)	2030 (71)	2853	326 (11)	2527 (89)
Sahiwal	1880	522 (28)	1358 (71)	1880	188 (10)	1691 (90)
Sargoda	1904	500 (26)	1404 (74)	1904	155 (8)	1749 (92)

Lower the mother’s education higher the chances that a child is facing stunting. For example, a woman with low education has a higher likelihood of having stunted children (36% moderate and 15% severe) compared to highly educated (9% moderately stunted and 2% severely stunted). Children whose father is educated (13% moderate and 4% severe stunting) are less likely to be stunted compared to those children whose father is not educated (38% moderate and 17% severe stunting).

If a woman marries at age less than 18 years as compared to those marries after 36 years (29% of their children are moderately stunted and 11% severely stunted) is more likely to a borne stunted child. A significantly high number of children 30% living in rural areas are moderately stunted relative to 21% in urban areas, whereas about 12% severely stunted compared to 7% in urban areas. There are significant differences among the administrative divisions of the Punjab province. For example, Rawalpindi division has a significantly lower prevalence of moderate and severe stunting (16% moderate and 5% severe stunting) compared D. G. Khan, Bahawalpur and Multan (39, 35 and 29% moderate stunting and 19, 15 and 11% severe stunting) respectively. Every fourth child 25%, (1.3–2.7) in Lahore, the provincial capital of Punjab, is moderately stunted and every tenth child 9% (1.3–2.7), is severely stunted. Those households having unimproved sanitation facilities are highly likely to have more stunted children (sanitation: 37% versus 23%). Surprisingly, with improved water facilities, the likelihood of stunting increases (19% unimproved versus improved 28%). However, with unimproved water facility the sample households are less than 5%. Almost 39% (18%) of those children living in food insecure districts are moderately (severely) stunted relative to 29% (1%) in vulnerable (borderline) districts and 24% (8%) in food secure districts.

### Multivariate analysis results

Tables 2 and 3 present the results of the multilevel logistic regression analyses and the results are presented as Odds ratio *(OR)* with their 95% Confidence Interval *(95% CI)*. The *LR* test favored the multilevel logistic regression instead of conventional logistic regression, shown by the statistically significant *(LR χ*^*2*^ *= 1005.94, P < 0.001)* for moderate stunting and *(LR χ*^*2*^ *= 628.88, P < 0.001)* for severe stunting *(see* Tables 2 and 3*).*

**Table 2:** Multivariable Regression results of Multilevel models for moderate stunting, MICS Punjab-2014 (n = 25,066)

Moderate Stunting SD > −2				
Variables	Model 1^a^	Model 2^b^*Cohen’s d* & OR [95% CI]	Model 3^c^*Cohen’s d* & OR [95% CI]	Model4^d^*Cohen’s d* & OR [95% CI]
***Individual and Household Level Characteristics***				
**Child age group***(Reference Category 0–5 months)*				
6–11		(0.52) 2.58*** [1.9,3.5]	(0.53) 2.61*** [1.9,3.5]	(0.53) 2.61*** [1.9,3.5]
12–23		(1.09) 7.25*** [5.1,10.3]	(1.10) 7.37*** [5.2,10.4]	(1.10) 7.41***[5.2,10.5]
24–35		(1.01) 6.25*** [4.1,9.5]	(1.01) 6.26*** [4.1,9.5]	(1.01) 6.26*** [4.1,9.5]
36–47		(1.50) 15.36*** [7.3,32.5]	(1.50) 15.23*** [7.2,32.4]	(1.51) 15.54***[7.3,33.1]
48–59		(1.42) 13.28*** [6.5,27.2]	(1.41) 13.09*** [6.3,27.1]	(1.42) 13.33*** [6.4,27.6]
**Child Birth order***(Reference Category First Born)*				
2		(0.25) 1.57***[1.4,1.8]	(0.24) 1.55***[1.4,1.7]	(0.24) 1.55***[1.4,1.7]
3+		(0.63) 3.14***[2.4,4.1]	(0.62) 3.06***[2.3,4.0]	(0.62) 3.05***[2.3,4.0]
**Child Gender***(Reference Category Boy)*				
Girl		(0.07) 1.14***[1.1,1.2]	(0.07) 1.14***[1.1,1.2]	(0.07) 1.14***[1.1,1.2]
**Child size at birth***(Reference Category Small Size)*				
Average		(0.27) 0.61***[0.45,0.81]	(0.27) 0.60***[0.46,0.80]	(0.27) 0.61***[0.46,0.81]
Large		(0.24) 0.65***[0.56,0.75]	(0.24) 0.64***[0.59,0.79]	0.24) 0.65***[0.55,0.76]
**Diarrhea***(Reference Category No)*				
Yes		(0.17) 0.73***[0.6,0.8]	(0.16) 0.74***[0.7,0.8]	(0.15) 0.75***[0.7,0.9]
**Breast Feeding***(Reference Category Yes)*				
No		(0.15) 1.35*[1.0,1.7]	(0.15) 1.36*[1.1,1.8]	(0.15) 1.35*[1.0,1.8]
**Mother Education***(Reference Category No Education)*				
Primary		(0.22) 0.67***[0.6,0.8]	(0.14) 0.77***[0.7,0.9]	(0.14) 0.77***[0.7,0.9]
Middle		(0.37) 0.51***[0.4,0.6]	(0.25) 0.63***[0.5,0.8]	(0.25) 0.64***[0.5,0.8]
Secondary		(0.42) 0.47***[0.4,0.6]	(0.24) 0.62***[0.5,0.8]	(0.24) 0.63***[0.5,0.8]
Higher		(0.76) 0.25***[0.2,0.3]	(o.59) 0.34***[0.3,0.4]	(0.59) 0.34***[0.3,0.4]
**Mother Age***(Reference Category age of women < 18 years of age)*				
18–24		(0.03) 0.94 [0.7,1.2]	(0.005) 0.99 [0.8,1.2]	(0.005) 0.99 [0.8,1.3]
25–35		(0.04) 0.93 [0.8,1.1]	(0.02) 0.97 [0.8,1.2]	(0.02) 0.97 [0.8,1.2]
36–49		(0.08) 0.86 [0.7,1.0]	(0.07) 0.88 [0.7,1.1]	0.07) 0.88 [0.7,1.1]
**Postnatal Care***(Reference Category Yes)*				
No		(0.02) 0.97 [0.9,1.1]	(0.02) 0.98 [0.9,1.1]	(0.02) 0.97 [0.9,1.1]
**Prenatal Care***(Reference category 1–4 visits)*				
More than 4 visit		(0.07) 0.88 [0.8,1.0]	(0.03) 0.94 [0.8,1.1]	(0.03) 0.95 [0.8,1.1]
**Delivery Place***(Reference category Home)*				
Hospital		(0.04) 0.93 [0.8,1.1]	(0.01) 0.98 [0.8,1.1]	(0.01) 0.98 [0.8,1.1]
**Father Education***(Reference category No Education)*				
Primary		(0.09) 0.85*[0.7,1.0]	(0.06) 0.90 [0.8,1.0]	(0.06) 0.91 [0.8,1.0]
Middle		(0.24) 0.65***[0.6,0.7]	(0.18) 0.71***[0.6,0.8]	(0.18) 0.72***[0.6,0.8]
Secondary		(0.32) 0.56***[0.5,0.6]	(0.23) 0.64***[0.5,0.7]	(0.24) 0.65***[0.6,0.8]
Higher		(0.50) 0.40***[0.3,0.5]	(0.40) 0.48***[0.4,0.6]	(0.40) 0.48***[0.4,0.6]
**Wealth index***(Reference category Poor)*				
Middle			(0.23) 0.64*** [0.7,0.8]	(0.24) 0.65*** [0.6,0.8]
Rich			(0.41) 0.47*** [0.5,0.6]	(0.40) 0.48*** [0.4,0.6]
**HH size***(Reference category HH size 3–4 members)*				
5–6 members			(0.05) 1.11 [1.0,1.4]	(0.05) 1.10 [1.0,1.3]
7–8 members			(0.11) 1.23* [1.0,1.5]	(0.11) 1.22* [1.0,1.4]
> 8 members			(0.09) 1.19 [1.0,1.5]	(0.08) 1.17 [1.0,1.4]
***Community Level Characteristics***				
**Ethnicity***(Refernececategory Urdu)*				
Punjabi			(0.08) 0.86 [0.7,1.1]	(0.08) 0.87 [0.7,1.1]
Saraikai			(0.03) 0.95 [0.7,1.2]	(0.06) 0.90 [0.7,1.2]
Others			(0.04) 1.08 [0.8,1.4]	(0.04) 1.08 [0.8,1.4]
**Region***(Reference category Urban)*				
Rural		(0.11) 1.22*** [1.1,1.3]	(0.03) 0.93 [0.9,1.0]	(0.03) 0.94 [0.9,1.0]
**Sanitation facility***(Reference category Unimproved)*				
Improved			(0.08) 0.86* [0.7,1.0]	(0.08) 0.87* [0.8,1.0]
**Drinking water facility***(Reference category Unimproved)*				
Improved			(0.07) 1.13 [0.7,1.5]	(0.07) 1.12 [0.8,1.5]
**Water treatment***(Reference category Treated)*				
Untreated			(0.13) 1.26 [1.0,1.7]	(0.13) 1.25 [1.0,1.6]
***Regional LevelCharacteristics***				
**Food Insecurity index***(Reference category Food Insecure)*				
Food Vulnerable				(0.14) 0.77* [0.6,0.9]
Food Secure				(0.19) 0.71*** [0.6,0.9]
**Division***(Reference category Rawalpindi Division)*				
Bahawalpur				(0.24) 1.56* [1.1,2.2]
D.G Khan				(0.23) 1.51* [1.0,2.2]
Faisalabad				(0.20) 1.45** [1.1,1.9]
Gujranwala				(0.19) 1.41* [1.1,1.9]
Lahore				(0.35) 1.88*** [1.3,2.7]
Multan				(0.19) 1.41* [1.1,1.9]
Sahiwal				(0.18) 1.39* [1.0,1.9]
Sargodha				(0.07) 1.13 [0.8,1.6]
Constant	−1.43 (0.13)***	0.038 (0.02)***	0.040 (0.019)***	0.030 (0.010)***
Level-1 (Household)	1.18 (0.10)***	2.19 *** (1.2,3.7)	2.17*** (1.3,3.7)	2.18*** (1.3,3.7)
Level-2 (Community)	0.53 (0,03)***	0.15 *** (0.08, 0.3)	0.14*** 0.07,0.3)	0.14*** (0.07,0.3)
Level-3 (District)	0.37 (0.03)***	0.04 *** (0.02,0.1)	0.03*** (0.01,0.08)	0.004 (0.0,0.02)
Chi2	(1005.94)***			

*Cohen’s d values are on the left side of each column in parenthesis*

OR = Odds Ratio, CI = 95% Confidence Intervals in brackets. * p < 0.05, ** p < 0.01, *** p < 0.001

*a = base model (unconditional three level hierarchical model), b = hierarchical model with child and parental characteristics, c = hierarchical model with child, parental and household characteristics and d = hierarchical model with child, parental, household and districts food insecurity index and division*

**Table 3:** Multivariable Regression results of Multilevel models for severe stunting, MICS Punjab-2014 (*n* = 25,066)

Severe Stunting SD > − 3				
Variables	Model 1^a^	Model 2b *Cohen’s d* & OR [95% CI]	Model 3^c^*Cohen’s d* & OR [95% CI]	Model4^d^*Cohen’s d* & OR [95% CI]
***Individual and Household Level Characteristics***				
**Child age group (months)***(Reference Category 0–5 months)*				
06–11		(0.24) 1.56 [1.9,3.5]	(0.24) 1.57 [1.0,2.6]	(0.24) 1.57 [1.0,2.6]
12–23		(0.87) 4.85***[5.1,10.3]	(0.88) 4.92***[2.9,8.2]	(0.88) 4.94***[3.0,8.2]
24–35		(0.84) 4.62***[4.1,9.5]	(0.84) 4.61***[2.5,8.7]	(0.84) 4.61***[2.5,8.7]
36–47		(1.02) 6.36***[7.3,32.5]	(1.02) 6.35***[2.3,17.9]	(1.02) 6.42***[2.3,18.3]
48–59		(1.07) 6.98***[6.5,27.2]	(1.07) 6.97***[2.4,20.3]	(1.08) 7.04***[2.4,20.7]
**Child order***(Reference Category First Born)*				
Second		(0.31) 1.76*** [1.4,1.8]	(o.30) 1.73*** [1.4,2.1]	(0.30) 1.72*** [1.4,2.1]
More than 2		(0.55) 2.71*** [2.4,4.1]	(0.53) 2.62*** [1.7,4.0]	(0.53) 2.60*** [1.7,3.9]
**Gender of child***(Reference Category Boy)*				
Girl		(0.13) 1.27***[1.1,1.2]	(0.13) 1.27***[1.2,1.4]	(0.13) 1.27***[1.2,1.4]
**Child size at birth***(Reference Category Small Size)*				
Average		(0.20) 0.69**[0.5,0.9]	(0.20) 0.70**[0.5,0.9]	(0.20) 0.68**[0.5,0.9]
Large		(0.25) 0.63***[.4,0.8]	(0.26) 0.62***[0.4,0.8]	(0.27) 0.61***[.4,.8]
**Diarrhea***(Reference Category No)*				
Yes		(0.25) 0.64***[0.6,0.8]	(0.25) 0.65***[0.6,0.8]	(0.25) 0.65***[0.6,0.8]
**Breast Feeding***(Reference Category yes)*				
No		(0.34) 1.87**[1.0,1.7]	(0.340 1.89**[1.3,2.8]	(0.340 1.87**[1.3,2.8]
**Mother Education***(Reference Category No Education)*				
Primary		(0.28) 0.60***[0.6,0.8]	(0.18) 0.72**[0.6,0.9]	(0.18) 0.72**[0.6,0.9]
Middle		(0.43) 0.46***[0.4,0.6]	(0.28) 0.61***[0.5,0.8]	(0.28) 0.61***[0.5,0.8]
Secondary		(0.50) 0.40***[0.4,0.6]	(0.32) 0.56***[0.4,0.7]	(0.32) 0.56***[0.4,0.7]
Higher		(0.83) 0.22***[0.2,0.3]	(0.64) 0.31***[0.2,0.5]	(0.64) 0.31***[0.2,0.5]
**Mother Age (years)***(Reference Category age of women < 18 years of age)*				
Age > =18 & < =24		(0.005) 0.99 [0.7,1.2]	(0.02) 1.03 [0.8,1.3]	(0.02) 1.03 [0.8,1.3]
Age > 24 & < =35		(0.06) 0.89 [0.8,1.1]	(0.04) 0.93 [0.7,1.2]	(0.04) 0.93 [0.7,1.2]
Age > 36		(0.04) 0.92 [0.7,1.0]	(0.04) 0.93 [0.7,1.2]	(0.04) 0.93 [0.7,1.2]
**Postnatal Care***(Reference category yes)*				
No		(0.03) 0.95 [0.9,1.1]	(0.03) 0.95 [0.8,1.2]	(0.03) 0.94 [0.8,1.2]
**Prenatal Care***(Reference category 1–4 visits)*				
More than 4 visit		(0.005) 0.99 [0.8,1.0]	(0.03) 1.06 [0.9,1.3]	(0.03) 1.07 [0.9,1.3]
**Delivery place***(Reference category Home)*				
Hospital		(0.03) 0.96 [0.8,1.1]	(0.02) 1.02 [0.8,1.3]	(0.02) 1.03 [0.8,1.3]
**Father education***(Reference category No Education)*				
Primary		(0.12) 0.80*[0.7,1.0]	(0.08) 0.86 [0.7,1.0]	(0.08) 0.87 [0.7,1.0]
Middle		(0.32) 0.56***[0.6,0.7]	(0.26) 0.63***[0.5,0.7]	(0.25) 0.64***[0.6,0.8]
Secondary		(0.32) 0.56***[0.5,0.6]	(0.23) 0.66***[0.5,0.8]	(0.23) 0.67**[0.5,0.9]
Higher		(0.49) 0.41***[0.3,0.5]	(0.38) 0.50***[0.4,0.6]	(0.37) 0.51***[0.4,0.6]
**Wealth Index***(Reference category Poor)*				
Middle			(0.27) 0.61*** [0.5,0.7]	(0.27) 0.61*** [0.5,0.7]
Rich			(0.43) 0.46*** [0.4,0.6]	90.43) 0.46*** [0.4,0.6]
**HH size***(Reference category HH size 3–4 members)*				
5–6 members			(0.09) 1.18 [0.9,1.5]	(0.09) 1.18 [0.9,1.5]
7–8 members			(0.11) 1.23 [0.9,1.6]	(0.11) 1.23 [0.9,1.6]
> 8 members			(0.11) 1.23 [1.0,1.6]	(0.11) 1.23 [1.0,1.6]
***Community Level Characteristics***				
**Ethnicity***(Reference category Urdu)*				
Punjabi			(0.09) 0.85 [0.5,1.3]	(0.09) 0.85 [0.5,1.3]
Saraikai			(0.07) 1.15 [0.8,1.7]	(0.06) 1.11 [0.7,1.7]
Others			(0.08) 1.17 [0.8,1.8]	(0.09) 1.18 [0.8,1.8]
**Region***(Reference category Urban)*				
Rural		(0.09) 1.18 [1.0,1.4]	(0.09) 0.85 [0.7,1.0]	(0.09) 0.87 [0.7,1.0]
**Sanitation facility***(Reference category Unimproved)*				
Improved			(0.13) 0.79** [0.7,0.9]	(0.13) 0.79** [0.7,0.9]
**Drinking water facility***(Reference category Unimproved)*				
Improved			(0.08) 1.17 [0.9,1.6]	(0.07) 1.15 [0.9,1.5]
**Water treatment***(Reference category Treated)*				
Untreated			(0.07) 1.15 [0.7,1.8]	(0.07) 1.17 [0.8,1.8]
***Regional Level Characteristics***				
**Food Insecurity index***(Reference category Food Insecure)*				
Food Vulnerable				(0.08) 0.87 [0.6,1.2]
Food Secure				(0.14) 0.77 [0.6,1.1]
**Division***(Reference category Rawalpindi Division)*				
Bahawalpur				(0.25) 1.57* [1.0,2.4]
D.G Khan				(0.34) 1.84** [1.2,2.9]
Faisalabad				(0.27) 1.63* [1.1,2.4]
Gujranwala				(0.26) 1.60* [1.0,2.5]
Lahore				(0.38) 1.98** [1.2,3.2]
Multan				(0.19) 1.41 [0.9,2.1]
Sahiwal				(0.25) 1.57* [1.1,2.3]
Sargodha				0.93 [0.6,1.4]
Constant	−3.14 (0.25) ***	0.011 (0.005) ***	0.013 (0.010) ***	0.012 (0.001) ***
Level-1 (Household)	1.16 (0.16) ***	2.51 *** (1.3,4.7)	2.50***(1.3,3.7)	2.50***(1.3,4.7)
Level − 2 (community)	0.55 (0.05) ***	0.20 ***(0.1, 0.4)	0.19*** (0.07,0.3)	0.19***(0.09.0.4)
Level − 3 (District)	0.48 (0.06) ***	0.10 ***(0.04,0.2)	0.06***(0.01,0.08)	0.02*(0.0,0.05)
Chi2	(628.88)***			

*Cohen’s d values are on the left side of each column in parenthesis*

OR Odds Ratio, CI = 95% Confidence Intervals in brackets. * p < 0.05, ** p < 0.01, *** p < 0.001

*a = base model (unconditional three level hierarchical model), b = hierarchical model with child and parental characteristics, c = hierarchical model with child, parental and household characteristics and d = hierarchical model with child, parental, household and districts food insecurity index and division*

Model 2 in Table 2 presents the results of only child specific and parent’s level characters and their association with moderate stunting. For example, compared to the reference category of zero to 5 months, children of 6 to 11 months are 2 and half times more likely to be stunted (OR = 2.58, CI = 1.9–3.5), whereas children of age 12–23 months are 7 times more prone to be stunted (OR = 7.25 (5.1,10.3). Girls are significantly more likely to be stunted than boys (OR = 1.14, 95% CI = 1.1–1.2). Children whose birth order is second is significantly likely to be stunted (OR = 1.57 95% CI = 1.4–1.8) whereas children whose birth order is 3 or more is 3 times more likely to be stunted (OR = 3.14 95% CI = 2.4–4.1). Children with no breastfeeding are not significantly likely to be moderately stunted (OR = 1.35 95% CI = 1–1.7) than those children who had breastfeeding. Children with no diarrhea are significantly less likely to be stunted compared to those having an episode of diarrhea (OR = 0.73 95% Cl = 0.6–0.8). Children whose size at birth perceived as average compared to small size at birth are less likely to be moderately stunted. (OR = 0.61 95% CI = 0.45–0.81). Mothers’ education is a significant covariate of moderate stunting. Compared to no education, reference category, children of highly educated mothers are significantly less likely to be moderately stunted (OR = 0.25 95% CI = 0.2–0.3). Whereas children of Primary (OR = 0.67 95% CI = 0.6–0.8) and Middle school completed mothers (OR = 0.51 95% CI = 0.4–0.6) are significantly less likely than no educated mothers to be stunted. Children of a father who has higher education are less likely to be stunted (OR = 0.40 95% CI = 0.3–0.5). Living in rural areas is a risk factor of moderate stunting. The results of severe stunting (less than minus 3 SD) are presented in Table 3.

Table 4 presents the results of *VPC* and *ICC* for both moderate and severe stunting. The total variance *(unconditional model 1)* by combining the variation at three levels (district, community and household) is estimated to be 5.37 (= 0.37 + 0.53 + 1.18 + 3.29)^2^ for moderate stunting. The district level explains 7% (=0.37/5.37) proportional change in variance *(VPC),* the community level variance shows 10% (=0.53/5.37) of variation and the household level variation implies that the risk of stunting varies significantly by 0.22% (=1.18/5.37). Similarly, looking at the *ICC* statistics of moderate stunting *(unconditional model 1),* we find that the district and community level *ICC* is 0.07 and 0.17 respectively. Thus, the correlation between two households from the same district, but different community is 0.07, while the correlation between two households from the same district and same community is higher at 0.17. This means that households in the same community have higher chance of correlation than the households of adjacent communities. This also shows that variance in the odds of a child being stunted is explained 7% by district and 17% by community characteristics.

**Table 4:** **Results from random intercept model: Measures of variation Variance Partition Coefficient (VPC) and Inter Class Correlation (ICC)**

Models	Model 1		Model 2		Model 3		Model 4	
***Standard Deviation***	−2 SD	−3 SD	−2 SD	− 3 SD	−2 SD	− 3 SD	-2 SD	-3 SD
***Variance Partition Coefficient (VPC)***								
*VPC*_*hh*_ = $\frac{\sigma_{hh}^2}{\sigma_{distt}^2+{\sigma}_{comm}^2+{\sigma}_{hh}^2+\uppi 2/3}$	0.22	0.21	0.37	0.41	0.38	0.40	0.38	0.42
*VPC*_*comm*_ = $\frac{\sigma_{comm}^2}{\sigma_{distt}^2+{\sigma}_{comm}^2+{\sigma}_{hh}^2+\uppi 2/3}$	0.10	0.10	0.03	0.03	0.04	0.03	0.03	0.03
*VPC*_*distt*_ = $\frac{\sigma_{distt}^2}{\sigma_{distt}^2+{\sigma}_{comm}^2+{\sigma}_{hh}^2+\uppi 2/3}$	0.07	0.08	0.007	0.02	0.006	0.02	0.007	0.003
***Intra Class Correlation (ICC)***								
*ICC*_*comm*_ = $\frac{\sigma_{distt}^2+{\sigma}_{comm}^2}{\sigma_{distt}^2+{\sigma}_{comm}^2+{\sigma}_{hh}^2+\uppi 2/3}$	0.17	0.19	0.03	0.05	0.03	0.05	0.03	0.03
*ICC*_*distt*_ = $\frac{\sigma_{distt}^2}{\sigma_{distt}^2+{\sigma}_{comm}^2+{\sigma}_{hh}^2+\uppi 2/3}$	0.07	0.08	0.007	0.02	0.006	0.02	0.007	0.003

Note: total variance = ${\sigma}_{distt}^2+{\sigma}_{comm}^2+{\sigma}_{hh}^2$ + π2/3

In summary, the *VPC* and *ICC* statistics show a high degree of clustering in the Punjab stunting data. However, the majority of the variation in stunting both moderate and severe lies at the household level. The differences could be due to income inequality, access to the health care facilities, food insecurity of households. Hence, the findings imply that stark variation exists among household demographic and socio-economic characteristics. Therefore, for policy relevance, household level nutrition related policy should be prioritized, followed by community and districts level measures.

## Discussion

We have analyzed the determinants of under nutrition (stunting) in Punjab Pakistan, which include 25,066 children of less than 5 years using multilevel hierarchical models. Our results indicate that stunting and severe stunting is a daunting public health challenge of the province of Punjab, Pakistan, because almost a little more than one fourth (27.4%) of children are moderately stunted and 10% are severely stunted in 2014. This number is alarming. The key individual and household level covariates significantly associated with stunting and severe stunting are; age of the child, birth order of the child, being female, being smaller in size at birth, episode of diarrhea, breast feeding, mother and father education, and wealth status of the household. A number of studies done on Africa and South Asia found that the risk of stunting and severe stunting increases with an increase in the age of a child [21, 29, 40–42]. It is because stunting can start during pregnancy *(*in utero*)* and is confirmed by a study using WHO child malnutrition and growth database as the average *z score* of infants drop rapidly until the age of 2 years [43, 44]. Moreover, as the child grow, their needs for food and diverse diet increases as well as their requirements for calorie intake. Given the limited household resources, it is inevitable that with the growing body needs unfulfilled, stunting will result.

Child birth order is a significant risk factor for child health outcomes. The higher the birth order it is highly likely that the odds of a child being stunted increase 3 folds, which is in line with the studies from India and Africa [45–47]. For India, the study explains that it is due to son preference and other reasons that mother’s time allocation for child care is distributed among children in the household and competition for household food and non-food resources [47, 48]. Gender of a child is an important factor in stunting outcome; for example; Girls are facing a relatively higher burden of stunting. Girls have 27% higher odds of being severely stunted and 14% moderately stunted relative to boys. This finding is in line with the study from Nepal and Pakistan [49, 50] and from rural Thatta, Pakistan [31]^,^ but in contrast with findings from Bhutan and Bangladesh [5, 51]. This gender bias depicts the patriarchal nature of social setup, especially in Punjab, Pakistan. This is confirmed by a study of 61 countries ranking Pakistan second-highest desired sex ratio for boys [52]. Furthermore gender discrimination and patriarchy leave female sex behind and neglect their fundamental rights like food. The findings of our study showed that children with diarrhea and less breast feeding at the earlier stages are more likely to be stunted. Similarly, rural residence (only in model 2 for moderate stunting) and smaller than the average size at birth are more likely to be stunted. These results are also reported in other studies [48, 53–55]. Mother and Fathers’ higher education status is associated significantly with the less likelihood of a child of less than 5 years being stunted (moderate and severe) in Punjab, Pakistan. This result is in line with other studies from Asia and Africa [28, 34, 56–58]. Although the channels through which education of parents can affect child health outcomes are debated as it hypothesized that higher educated parents are relatively better endowed with (health care) knowledge and resources, relatively rich and better able to provide childcare [28, 59, 60].

There are significant differences among the administrative divisions of the Punjab province. For example, Rawalpindi division has a significantly lower prevalence of moderate and severe stunting and compared to Rawalpindi, a child living in Lahore is twice likely to be stunted (moderate and severe) which is a surprising result given the relative socio-economic development of Lahore division compared to other administrative divisions of the province. One possible explanation is due to its metropolitan nature. Lahore division is relatively expensive to bear household food and non-food expenditure, especially for households with limited resources. Another possible reason can be that due to urbanization (82% population is urban in Lahore division) and at the same time it is highly congested (3500 person per square in Lahore district one of the highest in Punjab province and average household size 7.2 persons) and polluted city (poor living and unhygienic conditions) of the province which is detrimental for food absorption and health especially of children of younger ages [34, 39]. This result also points towards nutritional inequalities that exist among the administrative divisions of the Punjab province. This area of research needs further in depth empirical investigation. Mother’s age at marriage, household size, pre and postnatal care, drinking water facility and region of residence are not significant predictors of moderate and severe stunting in any of the models estimated. However, communities with improved sanitation facilities have less likelihood of a child being moderately or severely stunted. This finding is in line with the previous results reported by other studies [10, 61, 62]. While analyzing the data from 172 Demographic and Health Surveys, the results showed that the likelihood of stunting in the household with sanitation facilities was relatively lower [63].

The difficulty of interpreting the OR has troubled many clinical researchers and epidemiologists for a long time (Chen et al. 2010). Hence, the transformation has been done based on Cohen (1988). The small effect indicates a weak association; the medium indicates a moderate association, and the large indicates the strong association between the two groups. The *Cohen’s d* statistic has shown clearly an improvement as some of the variables compared with the reference point has significant OR ratio indicated by less than 0.001 *p values*, however, they fall in the small (weak association) category indicated by *Cohen’s d* values less than 0.20 (Tables 2 and 3).

Our study has following limitation; as MICS data is cross sectional in nature hence we cannot interpret our results as causality. Second, we do not have data available for mother’s diet and nutrition status which is an important variable of child health status. Finally, the MICS data is provincially representative but results cannot be generalizable at national level. Nevertheless, our findings are robust and setting stage for debate and policy discussions in one of the largest province of Pakistan. Hence, this study is an important step in formulating informed public policy decisions. Furthermore, there is no such analyses exist at sub national level in Pakistan that uses provincially representative large data set of children less than 5 years to analyze predictors of stunting.

## Conclusion

The findings of this study confirm that the child specific, household, and district characteristics have reliable predicting power in the explanation of stunting (moderate and severe). Furthermore, the results also reveal that stark differences exist among household level, while at the community and district level, the variances decrease as we incorporate the explanatory variables. The findings of this study have strong policy implication, indicating focus should be on the household while making public policy. Nevertheless, policy makers should not forget the role of community (village) and district level factors in policy priorities.

## Data Availability

MICS data set is publically available online on the following link: http://mics.unicef.org/surveys

## Abbreviations

MICS: Multiple Indicator Cluster Survey
OLS: ordinary least square
PCA: Principal Component Analysis
SSUs: Secondary Sampling Units
UNICEF: United Nations Children's Fund
WASH: water, sanitation and hygiene
WHO: World Health Organization

## Footnotes

1 *If baby weighs 4500 g or more at birth, they are considered larger than normal. Low birth weight less than 2500 g (upto and including 2499 g). Very low birth weight less than 1500 g (upto and including 1499 g)*

2 In the latent variable method, the individual variance level is π2/3, that approximately is 3.29. That is why we have added it to the total variance.
